# Differential Regulation of Protective and Harmful Renin Transcripts by the cAMP/PKA/Ca^2+^-Pathway in Cardiac H9c2 Cells

**DOI:** 10.3390/cells15141281

**Published:** 2026-07-17

**Authors:** Philipp Lutze, Kristin Jahn, Heike Wanka, Bianka Grunow, Jörg Peters

**Affiliations:** 1 Institute of Physiology, University Medicine of Greifswald, 17495 Greifswald, Germany; philipp.lutze@med.uni-greifswald.de (P.L.); kristin.jahn@med.uni-greifswald.de (K.J.); heike.wanka@med.uni-greifswald.de (H.W.); grunow@fbn-dummerstorf.de (B.G.); 2 Fish Growth Physiology Workgroup, Research Institute for Farm Animal Biology (FBN), 18196 Dummerstorf, Germany

**Keywords:** H9c2 cells, cAMP/PKA/Ca^2+^ signaling, renin-b, renin-a, glucose starvation

## Abstract

**Highlights:**

**What are the main findings?**

- In cardiac H9c2 cells, cAMP/PKA contributes to basal transcription of both renin-a and renin-b, but forskolin restores renin expression even during PKA inhibition, indicating additional PKA-independent cAMP effects, particularly on renin-b.
- In contrast to renal juxtaglomerular cells, intracellular Ca^2+^ does not inhibit but selectively stimulates renin-b expression in cardiac cells, especially under glucose starvation.

**What are the implications of the main findings?**

- The divergent Ca^2+^-dependent regulation of renin-b in cardiac versus renal cells supports the existence of an independent, tissue-specific renin system in the heart with potentially distinct physiological roles.
- Since both β-adrenergic/cAMP signaling and renin-b expression are upregulated during myocardial infarction, the identified cAMP/PKA/Ca^2+^-dependent pathway may contribute to the previously described cardioprotective effects of renin-b.

**Abstract:**

Two different renin isoforms are expressed in extrarenal tissues. The classical renin-a has been associated with detrimental effects, whereas renin-b exerts protective effects during glucose starvation. Glucose starvation selectively increased renin-b mRNA levels. 8Br-cAMP increased renin-a mRNA levels independently of glucose as well as of renin-b in glucose-starved cells. Adenylyl cyclase (AC) stimulation by forskolin increased expression of both renin transcripts, while AC inhibition by SQ22536 produced the opposite effect. PKA inhibition by KT5720 reduced the mRNA levels of both renin transcripts glucose-independently. Forskolin reversed the effect of KT5720 on renin mRNA levels. A23187-mediated increase in [Ca^2+^]i increased renin-b mRNA levels in glucose-starved cells. Ca^2+^ chelator BAPTA decreased renin-a mRNA expression in control cells and renin-b levels glucose-independently. Forskolin reversed the BAPTA-mediated decreases in renin-a but not renin-b expression. While the regulation of renin transcript levels by cAMP and PKA resembled known regulation in the kidney, the effect of intracellular free Ca^2+^ levels were opposite. This supports the existence of a separate renin system in cardiac cells.

## 1. Introduction

The renin–angiotensin system (RAS) plays a central role in regulating sodium as well as fluid balance and controls systemic blood pressure. The activity of the RAS is determined by the rate-limiting aspartyl protease renin, which is secreted predominantly by juxtaglomerular cells of the kidney and cleaves angiotensin (ANG) I from angiotensinogen. ANG°I is then processed by the angiotensin converting enzyme to form the effector peptide ANG°II. Given the pro-hypertrophic, pro-inflammatory, and pro-fibrotic effects of ANGII, the regulation of renin expression is of fundamental importance for the development of hypertension and heart and kidney failure [1,2,3,4,5,6]. Extensive data on the mechanisms that control renin gene expression are available for the kidney. Critical DNA sequences containing the proximal promotor and distal enhancer have been identified [7,8]. In juxtaglomerular renal cells, renin gene expression is regulated by G-protein coupled receptors (GPCR) that mediate the activation of protein kinase C (PKC) or adenylyl cyclase (AC) pathways. While the activation of PKC by the binding of ANG°II to its Gq-coupled ANG°II type 1 (AT1)-receptor causes inhibition of renin gene expression representing a negative feedback mechanism [9], the activation of Gs-regulated receptors and the subsequent AC/cyclic adenosine monophosphate (cAMP)/protein kinase A (PKA) signaling pathway increases renin gene expression. The increase in renin transcript level is mediated by accumulated cAMP, which either activates PKA and subsequently phosphorylates the cAMP response element-binding-protein (CREB) [10] or, similar to other binding proteins, binds to the 3′-untranslated region (UTR) of renin mRNA leading to its stabilization [11,12,13].

In addition to the circulating RAS, which is determined by renal renin secretion, there are also local RAS in extrarenal tissues. These systems are partially independent of the circulating system. For example, renin expression in the adrenal gland, as in the kidney, is markedly inhibited by ANGII. In contrast, renin secretion is stimulated by ANGII and Ca^2+^, factors known to inhibit renin secretion in the kidney [14,15]. Moreover, an additional renin transcript has been discovered in extrarenal tissues such as the adrenal gland and heart, which codes for a non-secretory, cytosolic renin called renin-b (previously (Exon1A-9)renin) [15,16,17]. In humans, renin-c also appears to exist [18]. Interestingly, the expression of renin-b transcripts increases in cardiac cells following myocardial infarction [16]. Furthermore, the overexpression of renin-b in cardiac cells has protective effects in cases of glucose depletion or hypoxia, reducing infarct size, necrosis, and apoptosis. At the cellular level, renin-b has been shown to reduce necrotic and apoptotic cell death and to lower reactive oxygen species accumulation under glucose and oxygen deprivation, effects that have been linked to the preservation of the mitochondrial membrane potential and ATP levels and that occur independently of angiotensin generation. In contrast to secretory renin-a, whose angiotensin-mediated actions promote hypertrophy, fibrosis, and cell death, renin-b thus represents a functionally distinct, stress-inducible isoform of the same gene [19,20,21].

The transcription of renin-b occurs from an alternative promotor located within intron A of the renin gene [22]. The transcript consists of an alternative 5′UTR and lacks exon 1, and translation begins within exon 2 of the known renin gene. The lack of exon1 results in the loss of the signal sequence required for co-translational transport to the endoplasmic reticulum (ER), as well as the first third of the pro-segment. Translation starts at the next AUG in exon 2, which is within the reading frame. The protein is a truncated prorenin with partial enzymatic activity that cannot be secreted and remains intracellular [15,17,18].

It is currently unknown whether there are specific regulatory elements in the renin promotor that influence or regulate the production of certain renin isoforms in response to physiological and pathophysiological signals. As demonstrated by Lutze et al. [22], in cardiac H9c2 cells, the TATA-less promoter upstream of alternative exon1A is regulated by glucose starvation in a serum response factor (SRF)-dependent manner. Given the role of cAMP in the renal regulation of renin-a transcript rates, and considering the chronic activation of β-adrenoreceptors during myocardial infarction, which leads to increased cAMP production [23], we investigated here the significance of the AC/cAMP/PKA/Ca^2+^ signaling in the regulation of renin-a and renin-b transcription in response to glucose starvation in cardiac H9c2 cells.

## 2. Materials and Methods

### 2.1. Reagents

8-Bromo-cAMP (5 mmol/L), forskolin (adenylyl cyclase activator; 10 µmol/L), SQ22536 (adenylyl cyclase inhibitor; 100 µmol/L), 1,2-Bis(2-aminophenoxy)ethane-N,N,N′,N′-tetraacetic acid tetrakis acetoxymethyl ester (BAPTA, chelator of intracellular Ca^2+^ stores, 50 µmol/L), and A23187 (calcium ionophore, 1 µmol/L) were obtained from Sigma-Aldrich (Taufkirchen, Germany). KT5720 (protein kinase A inhibitor, 5 µmol/L) was obtained from Tocris Bioscience (Bristol, UK).

### 2.2. Cell Culture

H9c2 cells (ATCC, Manassas, VA, USA) were maintained in Dulbecco Modified Eagle medium (DMEM) (Lonza, Basel, Switzerland) supplemented with 10% fetal bovine serum (PAN Biotech, Darmstadt, Germany), 100 U/mL penicillin, and 100 µg/mL streptomycin (GIBCO, Life Technologies, Darmstadt, Germany) in a humidified incubator at 5% CO_2_ and 37 °C. For PCR analyses, 2 × 10^5^ cells were seeded in 6-well culture plates (Sarstedt AG, Nümbrecht, Germany) in 2 mL complete DMEM for 3 days. The cells were then exposed to stimulators or inhibitors of the adenylate cyclase pathway for 24 h under both control conditions and glucose depletion. Control conditions corresponded to standard DMEM containing 25 mmol/L glucose, while glucose starvation was performed in otherwise identical DMEM containing 0 mmol/L glucose; glutamine and pyruvate were present at standard concentrations in both conditions. The two media were matched in composition except for glucose. The compounds were dissolved in DMSO (Carl Roth, Karlsruhe, Germany) or water as appropriate; the respective solvents had been tested in preliminary experiments and showed no effect on cell viability or renin transcript levels. After trypsination and washing in phosphate-buffered saline buffer (PBS) (PanBiotech, Aidenbach, Germany), the cells were counted and cell viability was determined using the trypan blue exclusion test. The cell pellets were stored at −20 °C until RNA extraction was performed.

### 2.3. Quantitative Real-Time PCR

RNA was extracted using the RNeasy Mini Kit (Zymo Research, Freiburg, Germany) according to the manufacturer’s instructions. The quality was checked using a spectrophotometer (DS-11+, DeNovix Inc., Wilmington, DE, USA). The RNA was reverse transcribed into cDNA using the High Capacity cDNA Kit (Life Technologies, Darmstadt, Germany) and stored at −70 °C. For qRT-PCR, the cDNA was diluted in nuclease-free water. Duplicates of 20 ng cDNA were then mixed with SYBR^®^ FAST Universal 2X qPCR Master Mix (Biocompare, San Francisco, CA, USA), which contained SYBR green dye and optimised primer pairs, as shown in Table 1. To calculate the gene expression levels of renin-a and renin-b, the threshold cycle number (CT) was normalised against the housekeeper YWHAZ using the 2^−∆CT^ method. YWHAZ was selected as the reference gene based on preliminary testing, in which its expression was not significantly regulated under glucose starvation. Each biological replicate was measured in technical duplicates, which were averaged prior to analysis; n refers throughout to the number of independent biological experiments.

### 2.4. Determination of ATP and cAMP Levels

H9c2 cells (10^4^/well) were cultured for 72 h in 96-well plates (cellstar, Greiner BioOne, Krimsmünster, Austria) in a humidified atmosphere (5% CO_2_, at 37 °C) to allow the cells to adhere. The cells were then exposed to control conditions or glucose starvation for a further 24 h before ATP and cAMP levels were analysed.

The CellTiter-Glo^®^ Luminescent Cell Viability Assay was used to measure cellular ATP content in accordance with the manufacturer’s instructions (Promega, Mannheim, Germany). It based on the conversion of luciferin to oxyluciferin, pyrophosphate, and light in the presence of ATP. The amount of light as a measure of ATP content was determined using a microplate luminometer (Berthold, Bad Wildbach, Germany).

Cyclic AMP (cAMP) content was determined by using a commercially available ELISA kit (Enzo Life Science, Loerrach, Germany) according to the manufacturer’s instructions. Prior to analysis, the medium was removed and 0.1 mol/L HCl were added to the cells for 10 min at room temperature to induce cell lysis. When lysis was sufficient, the culture plate was centrifuged at 1000× *g* to pellet the cell debris. The supernatant was immediately examined according to the manufacturer’s instructions. The cAMP content was calculated using the corresponding cAMP standard curve.

### 2.5. Measurement of Cytosolic Calcium Concentration

Cytosolic calcium (Ca^2+^) concentration was measured 24 h after exposure of H9c2 cells to control conditions or glucose starvation in combination with different test substances. Briefly, 10^5^ pretreated H9c2 cells were incubated for 45 min at room temperature in the dark with a 1:1 mixture of Fluo3-AM (2 µmol/L, Biotrend, Germany) and Pluronic F127 in RPMI medium (PanBiotech, Aidenbach, Germany) supplemented with 2% FCS and 2 mmol/L HEPES. After washing in 2 mL Hanks buffer, the cells were resuspended in Hanks buffer, incubated for a further 30 min at room temperature in the dark, and then analysed using the FACS Calibur flow cytometer and the Cell Quest software (BD Biosciences, Franklin Lakes, NJ, USA). The data are represented as the mean fluorescence intensity of Fluo3-AM-positive cells, which reflects the free Ca^2+^ content in the cytosol. Viable cells were identified by a live-cell gate based on forward scatter (FSC) and side scatter (SSC) characteristics, excluding cell debris. Within this gate, Fluo3-AM fluorescence was recorded, and the mean fluorescence intensity (MFI) of the Fluo3-AM signal was used as the readout of free cytosolic Ca^2+^. MFI values were compared between treatment conditions and the respective controls.

### 2.6. Statistical Analyses

Data are presented as mean ± SEM values. Statistical analyses were performed on ΔCT values, the 2^−ΔCT^ transformation was used only for graphical representation. Datasets involving two experimental factors were analysed by two-way ANOVA followed by Tukey’s post hoc test, while two-group comparisons were performed using Student’s *t*-test or one-way ANOVA followed by Tukey’s post hoc test, using GraphPad Prism software 8. Differences were considered statistically significant at *p* < 0.05.

## 3. Results

### 3.1. Differential Regulation of Renin Transcripts by cAMP and Glucose Starvation

**Figure 1:**
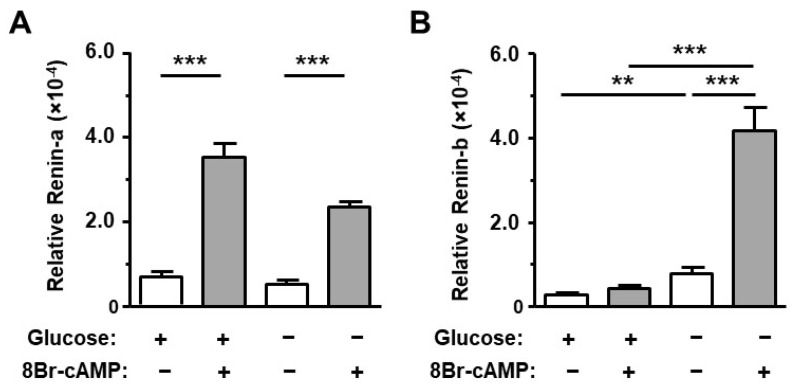
Effect of 8Br-cAMP on renin mRNA levels in H9c2 cells. Analyses were performed using H9c2 cells exposed to control conditions or glucose starvation in combination with or without 5 mmol/L 8Br-cAMP supplementation. Expression pattern of (**A**) renin-a mRNA and (**B**) renin-b mRNA levels were normalised to the housekeeper gene YWHAZ. Data represent mean ± SEM values from six individual experiments. Statistical analyses were performed by two-way ANOVA followed by Tukey’s post hoc test with ** *p* < 0.01, *** *p* < 0.001.

In kidney cells, cAMP has been shown to be a positive regulator of classical renin-a transcription. Similarly, in cardiac H9c2 cells, renin-a transcription increased by application of 8Br-cAMP (Figure 1A). We found a 5.4-fold increase in renin-a levels under basal conditions and a 4.4-fold increase in H9c2 cells subjected to glucose starvation. Basal expression of renin-b was very low in this experiment, and an effect of 8Br-cAMP was not observed (Figure 1B). However, glucose starvation led to a selective increase in renin-b transcription. Thus, glucose-starved H9c2 cells showed a 4.7-fold increase in renin-b mRNA levels triggered by 8Br-cAMP.

**Figure 2:**
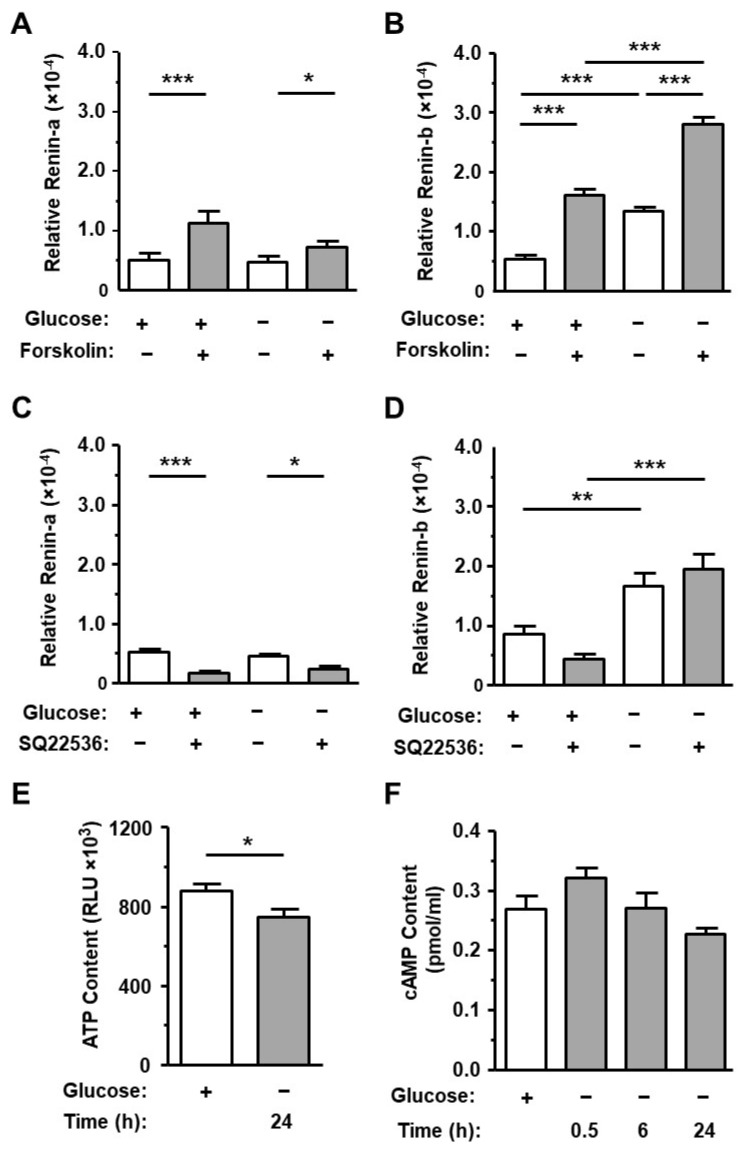
Effects of adenylate cyclase activation or inhibition on renin mRNA levels in H9c2 cells. H9c2 cells were exposed to basal conditions or glucose starvation in combination with various adenylyl cyclase (AC) modulators. Renin-a mRNA and renin-b mRNA levels were normalised to the housekeeping gene YWHAZ. (**A**,**B**) Effects of the AC activator forskolin (10 µmol/L) and (**C**,**D**) Effects of the AC inhibitor SQ22536 (100 µmol/L). (**E**,**F**) Effect of glucose deprivation on cellular ATP and cAMP levels at the indicated time points. Data represent mean ± SEM values of 6 independent experiments. Statistical analyses of the renin transcript data (**A**–**D**) were performed by two-way ANOVA followed by Tukey’s post hoc test; the ATP and cAMP data (**E**,**F**) were analysed by one-way ANOVA followed by Tukey’s post hoc test. * *p* < 0.05, ** *p* < 0.01, *** *p* < 0.001.

Under physiological conditions, cellular cAMP concentration is primarily determined by the concerted activities of adenylyl cyclases (AC), which produce cAMP, and phosphodiesterases (PDE), which degrade cAMP. To investigate the role of endogenously produced cAMP, we next analysed the effects of AC stimulation and inhibition on renin expression (Figure 2). Forskolin-induced stimulation of AC resulted in a 2.4-fold increase in renin-a mRNA levels under basal condition, whereas this effect was not found after glucose starvation (Figure 2A). Renin-b mRNA levels increased 3.5-fold under basal condition and 2.1-fold in glucose-starved H9c2 cells (Figure 2B).

Inhibition of AC by SQ22536 resulted in a significant 4-fold decrease in renin-a mRNA levels in H9c2 cells cultured under basal conditions as well as under glucose starvation (2-fold) (Figure 2C). Renin-b mRNA levels in SQ22536-treated cells tendentially decreased under basal conditions, while no inhibitory effect of the AC inhibitor on renin-b mRNA levels was detectable in glucose-starved H9c2 cells (Figure 2D). This suggests that cellular cAMP levels are crucial for the transcription of renin isoforms in cardiac H9c2 cells under basal conditions, but that cAMP is unlikely to mediate the increase in renin-b mRNA levels during glucose starvation.

Since glucose deficiency can be associated with a reduction in ATP production and, subsequently, in cAMP content, we analysed both parameters. In H9c2 cells, glucose starvation resulted in a significant decrease of cellular ATP levels (Figure 2E) but did not affect cellular cAMP content to any time point investigated (Figure 2F). This suggests that glucose starvation did not sufficiently deplete ATP levels to affect cAMP production in H9c2 cells.

### 3.2. PKA Contributes to Renin Gene Expression in Cardiac H9c2 Cells

**Figure 3:**
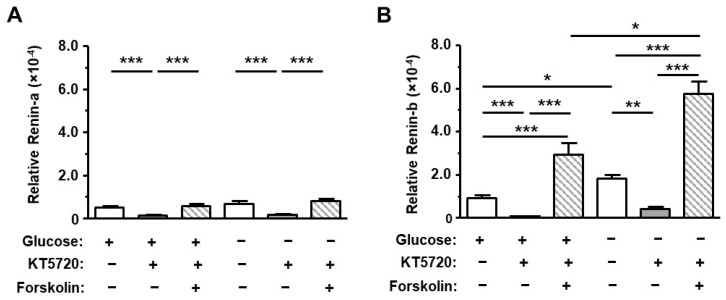
Effect of protein kinase A inhibition on renin expression in H9c2 cells. Analyses were performed using H9c2 cells exposed to basal conditions or glucose starvation in combination with the protein kinase A inhibitor KT5720 (5 µmol/L) and the AC activator forskolin (10 µmol/L), as indicated. The expression pattern of (**A**) renin-a and (**B**) renin-b mRNA were normalised to the housekeeping gene YWHAZ. Data represent mean ± SEM values of 7 independent experiments. Statistical analyses were performed by two-way ANOVA followed by Tukey’s post hoc test with * *p* < 0.05, ** *p* < 0.01, *** *p* < 0.001.

Given its known regulatory role in transcription of secretory renin, we next investigated the role of cAMP-regulated PKA in the regulation of both renin transcripts using the PKA inhibitor KT5720 (Figure 3). Regardless of culture conditions, we found a marked KT5720-induced downregulation of both renin-a and renin-b mRNA levels, consistent with a role of PKA in renin expression (Figure 3A,B).

Next, we examined whether the addition of forskolin could prevent KT5720-mediated downregulation of renin (Figure 3A,B). Indeed, renin-a transcript levels increased to levels similar to those of the corresponding controls without KT5720 treatment. In comparison, forskolin not only reversed the KT5720-mediated downregulation of renin-b but also increased renin-b transcription compared to the corresponding controls.

### 3.3. In Cardiomyoblasts Renin Expression Is Stimulated by Intracellular Free Ca^2+^ Ions

**Figure 4:**
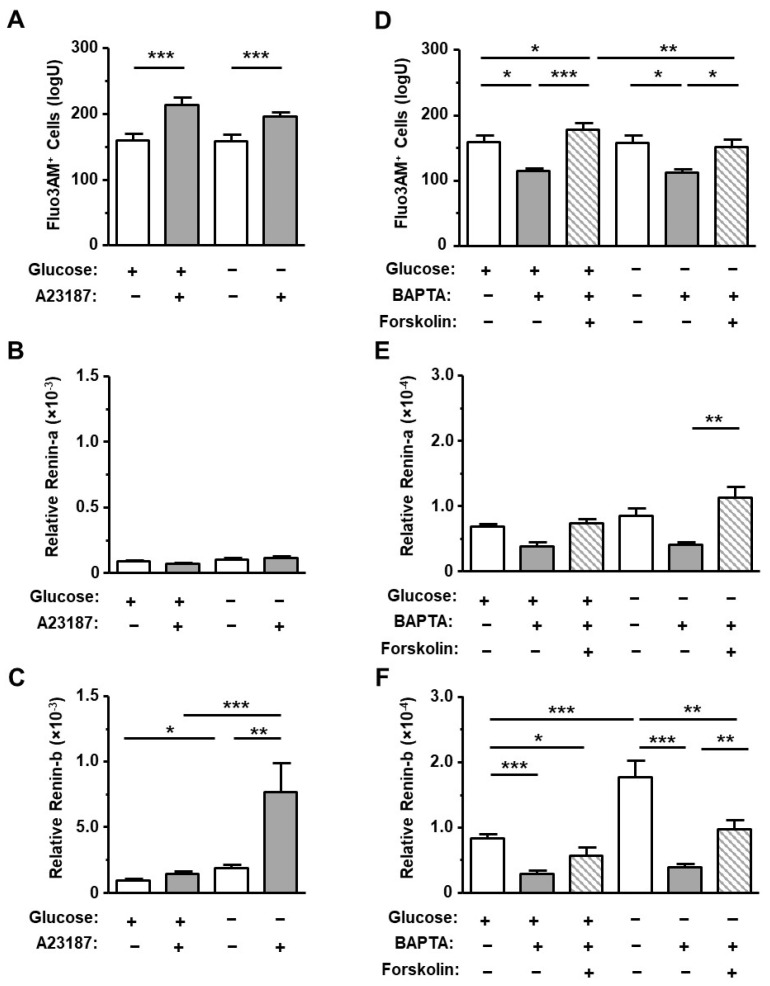
Renin expression in H9c2 cells is determined by the intracellular Ca^2+^ level. For analyses, H9c2 cells were exposed to basal conditions or glucose starvation alone or in combination with the calcium ionophore A23187 (1 µmol/L) or the Ca^2+^ chelator BAPTA (50 µmol/L) without or with forskolin (10 µmol/L) for 24 h. (**A**,**D**) The intracellular calcium content ([Ca^2+^]i) was monitored by flow cytometry using the Ca^2+^-sensitive fluorophore Fluo3AM (n = 7). The levels of (**B**,**E**) renin-a mRNA and (**C**,**F**) renin-b mRNA were normalised to the housekeeping gene YWHAZ. Data represent mean ± SEM values of 8 independent experiments. Statistical analyses were performed using two-way ANOVA followed by Tukey’s post hoc test with * *p* < 0.05, ** *p* < 0.01, *** *p* < 0.001 compared to the respective controls, as indicated.

In juxtaglomerular cells of the kidney, increases in intracellular Ca^2+^ concentration ([Ca^2+^]_i_) inhibit renin expression and secretion while a decrease of [Ca^2+^]_i_ stimulates both parameters. To clarify the function of [Ca^2+^]_i_ on cardiac renin expression, H9c2 cells were exposed to either the calcium ionophore A23187 or to the Ca^2+^ chelator BAPTA without or with forskolin supplementation (Figure 4 and Figure S1).

The free cytosolic [Ca^2+^] was quantified by the fluorescence intensity of Fluo3AM-labelled cells. As expected, the Ca^2+^ ionophore A23187 significantly increased the free [Ca^2+^]_i_ of control as well as glucose-starved cells (Figure 4A). Vice versa, BAPTA significantly decreased the free [Ca^2+^]_i_ in both groups. Simultaneous addition of forskolin corrected the BAPTA-induced decrease in [Ca^2+^]_i_ (Figure 4D).

The Ca^2+^ ionophore A23187 had no effects on renin-a mRNA expression under either basal or glucose starvation conditions (Figure 4B). In contrast, renin-b transcript levels were increased (Figure 4C). Thus, A23187 induced a moderate 1.9-fold increase in renin-b expression in control cells and a marked 4.1-fold increase in glucose-starved H9c2 cells.

BAPTA alone reduced the renin-a mRNA levels in controls and glucose-starved cells (Figure 4E). Forskolin reversed the BAPTA-mediated downregulation of renin-a. Thus, the renin-a mRNA levels increased significantly in glucose-starved cells to baseline levels. In comparison, the BAPTA-induced decrease of renin-b mRNA was more pronounced (Figure 4F). The mRNA levels of renin-b decreased by 2.9-fold in controls and even by 4.5-fold in glucose-starved cells. Again, forskolin reversed the BAPTA effects, but to a lesser degree.

## 4. Discussion

**Figure 5:**
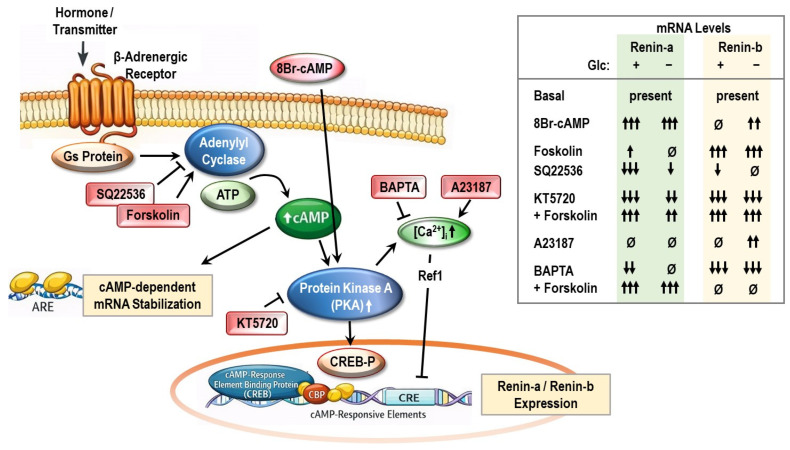
Influence of cAMP/PKA and Ca^2+^ signaling on the expression of renin-a and renin-b in cardiac H9c2 cells. In general, renin mRNA levels are regulated by the second messengers cAMP and Ca^2+^, which can affect both the renin expression itself and mRNA stabilization. The relevance of individual components of the signaling pathway was tested using specific inhibitors or stimulators of AC, PKA, and [Ca^2+^]_i_, as indicated. The results are summarised in the accompanying table with Ø without effects, ↑ significant increase, and ↓ significant decrease in renin mRNA levels.

The present study demonstrates the significance of the AC/cAMP/PKA and Ca^2+^ signaling for the regulated renin expression in cardiac H9c2 cells, where both secretory renin-a and cytosolic renin-b isoforms are constitutively expressed. As summarised in Figure 5, the data demonstrate that in H9c2 cells the transcript levels of renin-a are regulated by cellular cAMP level as well as by PKA activity, similar to what has been described for kidney’s secretory renin-a. Both AC and PKA activities are also essential for increasing renin-b transcription. Secondly, there are additional PKA-independent cAMP effects that selectively influence renin-b mRNA expression. Thirdly, in contrast to kidney cells, which respond to increased [Ca^2+^]i with decreased renin-a mRNA levels, cardiac H9c2 cells respond with an increase in renin-b mRNA levels. Although basal [Ca^2+^]i appears to be required for the expression of both renin transcripts, the effect of an increase in [Ca^2+^]i seems to be specific for renin-b, as renin-a did not respond to the Ca^2+^ ionophore A23187. The decrease of renin-a mRNA levels in H9c2 cells seen by BAPTA administration indicates an inverse regulation of renin-a compared with renal cells. Furthermore, in contrast to the known inhibitory effect of A23187-induced increase in [Ca^2+^]i in renal cells [9], A23187 did not at all decrease renin-a mRNA levels in H9c2 cells. Fourthly, the renin-b expression under glucose-starvation conditions seems to be subjected to a Ca^2+^-dependent regulation.

Intracellular cAMP levels depend on the stimulation of Gs and Gi protein-coupled receptors (GPCRs), the concerted activity of ACs and PDEs, and the availability of ATP. In our study, cAMP production under basal and glucose starvation conditions was not limited by ATP levels, as our cAMP and ATP measurements showed. In fact, the cAMP levels remained unchanged at all time points, despite a significantly reduced cellular ATP level after glucose starvation.

It is well-known that increase in intracellular cAMP levels stimulates PKA activation, followed by phosphorylation of cAMP-responsive element binding protein (CREB) or CREB-associated transcription factor 1 (ATF-1), leading to enhanced renin gene expression in JG cells [10,24,25]. Similarly, a cAMP-mediated increase in renin-a mRNA levels could occur in cardiac H9c2 cells. Computer-based analyses using the AliBaba2.1 program, however, showed that such cAMP-responsive elements (CREs) are not present in the promoter region of the alternative renin-b. Instead, various CCAAT/enhancer binding protein (C/EBP) binding sites could be identified. Hirai et al. [26] showed that the transcription of C/EBP-responsive genes can also be regulated via cAMP and CREB. Thus, forskolin-induced upregulation of renin-b expression may be mediated indirectly through cAMP/CREB-dependent activation of C/EBP transcription factors rather than through direct CRE-mediated transcription. However, this hypothesis requires experimental validation. The AC Inhibitor SQ 22536 markedly decreased both renin-a and renin-b transcript levels, confirming the relevance of cAMP signaling for renin expression in cardiac cells.

To decipher the role of PKA and to differentiate between direct cAMP effects and those triggered by the PKA, we used the PKA inhibitor KT5720 [27]. PKA inhibition by KT5720 suppressed both renin-a and renin-b expression, indicating that PKA activity is involved in basal renin transcription in H9c2 cells. However, the activation of AC by forskolin reversed or restored renin expression despite PKA inhibition by KT5720, suggesting that cAMP plays an additional role in renin expression independent of PKA activity. As KT5720, like most small-molecule kinase inhibitors, may exert off-target effects, including a more potent inhibition of phosphorylase kinase than of PKA[28], the involvement of PKA is supported here primarily by the convergence of adenylyl cyclase activation, inhibition, and forskolin-mediated rescue rather than by KT5720 alone. The activation of AC by forskolin reversed or restored renin expression despite PKA inhibition by KT5720, suggesting that cAMP plays an additional role in renin expression independent of PKA activity. In this case, the restoration of renin-b expression by forskolin during PKA inhibition was even more effective than that of renin-a. In this context, the regulation of renin expression includes not only transcription itself but also post-transcriptional mechanisms. Chen et al. [11] reported that, in JG cells, the transcription rate of renal renin-a increased 3.9-fold following treatment with forskolin and its half-life was prolonged from 3 to 10.8 h. Our preliminary data suggest that in H9c2 cells the regulation of mRNA stability is less pronounce, the half live of renin-b being shorter than of renin-a and with only a slight increase of renin mRNA half times by cAMP (Figure S2).

Such mechanisms are partially based on the expression of various RNA-binding proteins such as human antigen R (HuR), dynamin, nucleolin, hydroxyacyl-CoA dehydrogenase/3-ketoacyl-CoA thiolase/enoyl-CoA hydratase beta subunit (HADHB), or Y box binding protein 1 (YB1). The functionality of some of these proteins is regulated by cAMP and affects renin-a expression [12,13,29]. Their significance for renin-b expression, however, still needs to be clarified.

In addition to its role in regulating gene expression, PKA increases the intracellular Ca^2+^ concentration ([Ca^2+^]i) in the heart by phosphorylating L-type Ca^2+^ channels in the cell membrane and ER-localised Ca^2+^ channels (ryanodine receptor 2, RyR2). Such Ca^2+^ channels are also expressed in H9c2 cells [30,31]. In our study, KT5720-mediated inhibition of PKA as expected led to a reduction of [Ca^2+^]i, while concomitant substitution of forskolin restored it (Figure S1). To further investigate the role of free cytosolic calcium ions on renin expression, we used the calcium chelator BAPTA-AM and the calcium ionophore A23187 decreasing or increasing [Ca^2+^]i, respectively. In fact, the BAPTA-mediated decrease in [Ca^2+^]i efficiently reduced the expression of both renin transcripts, with a more pronounced effect on renin-b. This result suggests that in H9c2 cells, free calcium ions are required for basal renin-a and renin-b transcription. This assumption is supported by the observation that forskolin reversed both the BAPTA-mediated decrease in [Ca^2+^]i and the reduction in renin expression. In this context, Hardingham et al. [32] demonstrated that an increase in nuclear calcium concentration in hippocampal neurons, through the activation of calcium/calmodulin-dependent protein kinases, leads to the recruitment of CREB. CREB then acts as a nuclear, calcium-responsive transcription factor. Additionally, a second pathway activates transcription through the serum response element via rise in cytosolic calcium. The latter fact may account for the significant increase in renin-b expression in H9c2 cells simultaneously exposed to glucose starvation and the calcium ionophore A23187. In support, we demonstrated in a previous study that serum response factor is essential for renin-b expression during glucose starvation [22]. However, other studies suggest disruption of the cAMP signaling pathway by calcium. Such a link results from the existence of calcium-inhibitable ACs or calcium-activatable cAMP phosphodiesterase isoforms. These enzymes are known to contribute to the Ca^2+^ paradox of renin-a expression and release in renal JG cells [33,34]. Thus, increases of [Ca^2+^]i due to thapsigargin-induced blockade of the Ca^2+^ ATPase SERCA inhibited renin-a expression of As4.1 cells [9]. Since the calcium-inhibitable AC5 and AC6 are the most important AC isoforms in the heart [35], one could also expect an inhibition of renin expression through an increase in [Ca^2+^]i in cardiac cells. However, Ca^2+^ ionophore A23187-mediated increases in [Ca^2+^]i had no effects on renin-a expression in H9c2 cells. These discrepancies may be due to tissue-specific differences, the level of Ca^2+^ released into the cytosol, or the localization of intracellular calcium ions. In this context, Bers [36] described that the β-receptor/cAMP/PKA signaling cascade in the heart is relevant for local Ca^2+^-mediated effects occurring in closely restricted spheres, called microdomains. The authors suggested that such domains could also be involved in Ca^2+^-dependent regulation of transcription, which may explain why different modes increasing intracellular Ca^2+^ levels had divergent effects on renin expression. Another explanation could be that cytosolic calcium ions may be integrated in a fine-tuning machinery that modulates the direct influence of cAMP and/or PKA on renin expression. During the forskolin substitution, the direct effects of cAMP and PKA on renin expression dominate, while calcium ions have a modulatory effect by influencing the activity of ACs, PDEs, and renin expression itself. It is expected, that ANGII, which inhibits renin expression in the kidney and increases [Ca^2+^]I via the PLC/IP3 cascade, will also stimulate renin-b expression in cardiac cells.

Finally, in the light of the chronic activation of β-adrenoreceptors during myocardial infarction, we analysed the gene expression of renin after glucose starvation, which is part of the ischemic process during infarction. In fact, we found several differences, particularly in renin-b expression, between control and glucose starved cells. Similar as documented in former studies [19], the renin-b expression in glucose-starved H9c2 cells was increased compared to control cells. In addition, in glucose-starved H9c2 cells, only the renin-b expression increased significantly in response to cAMP or forskolin substitution, as well as after simultaneous PKA inhibition and AC activation. These results suggest a differential regulation of renin-a and renin-b under these conditions. In yeast, the cAMP/PKA pathway is indeed under the control of an intracellular and extracellular glucose sensing system [37]. In mammalian cells, however, other regulators are known to be involved in the control of cellular nutrient and energy levels such as AMP-activated protein kinase (AMPK) and the mTOR signaling pathway. An interaction with or by the PKA pathway has been described for both components [38,39]. Since the cAMP level remained unchanged during glucose starvation, we hypothesise that AMPK-mediated signaling and/or the mTOR pathway might directly modulate renin gene expression independently of cAMP/PKA/Ca^2+^ signaling. This assumption requires further investigation.

The present study focuses on the transcriptional regulation of the two renin isoforms. Their protein levels, enzymatic activity, and downstream functional consequences were not addressed in the present work, which represents a limitation with respect to directly demonstrating the functional roles of the two isoforms. Linking the transcriptional regulation described here to the corresponding protein and functional changes will be the subject of dedicated studies.

## 5. Conclusions

In cardiac H9c2 cells, the two renin transcripts encoded by the same gene are part of an endogenous regulatory system in which cAMP/PKA/Ca^2+^ signaling controls basal transcription of both renin-a and renin-b. This regulation differs in part from that described for juxtaglomerular cells of the kidney, indicating tissue-specific differences. During glucose starvation renin-b, but not renin-a is upregulated. Here, both cAMP/PKA/Ca^2+^-dependent and independent mechanisms may contribute to this induction. As cAMP/PKA/Ca^2+^ signaling and renin-b expression are both increased during myocardial infarction, β-adrenergic receptor activation may promote renin-b expression and thereby contribute to cardioprotection.

## Figures and Tables

**Table 1:** Primer sequences for the detection of gene expression.

Gene	Primer Sequence	
	Forward	Reverse
renin-b	TGAATTTCCCCAGTCAGTGAT	GAATTCACCCCATTCAGCAC
renin-a	ATGAATTCACCCCATTCAGC	CCAGATGGGCGGGAGGAGGATG
YWHAZ	CATCTGCAACGACGTACTGTCTCT	GACTGGTCCACAATTCCTTTCTTG

## Data Availability

The data presented in this study are available on request from the corresponding author.

## Supplementary Materials

The following supporting information can be downloaded at: https://www.mdpi.com/article/10.3390/cells15141281/s1, Figure S1. Effects of the calcium ionophore A23187 or the calcium chelator BAPTA on the free intracellular Ca^2+^ levels. For analyses, H9c2 cells were exposed to control conditions or glucose starvation alone or in combination with the calcium ionophore A23187 (1 µmol/L) or the calcium ions chelator BAPTA (50 µmol/L) without or with forskolin (10 µmol/L) for 24 h. The intracellular calcium content ([Ca^2+^]i) was monitored by flow cytometry using the Ca^2+^-sensitive fluorophore Fluo3AM. Data show representative FACS images that include the percentage of Fluo3AM-positive H9c2 cells and their mean fluorescence intensity (logU). Figure S2. Half-lives of renin-a and renin-b mRNAs. Analyses were performed on H9c2 cells exposed to glucose starvation without or with cAMP supplementation, as indicated. (A) Half-life of renin-a and (B) half-life of renin-b transcripts after inhibition of RNA synthesis by actinomycin D at the indicated time points. Data represent mean ± SEM values from 3 independent experiments. Table S1. Relative basal abundance of renin-a and renin-b transcripts in cardiac H9c2 cells. Transcript levels were determined by qRT-PCR and normalised to the reference gene YWHAZ (ΔCT). A lower ΔCT corresponds to higher transcript abundance. Values are given as mean ± standard deviation; n indicates the number of independent biological experiments.

## Abbreviations

The following abbreviations are used in this manuscript:

AC	adenylyl cyclase
RAS	renin-angiotensin system
GPCR	G-protein coupled receptors
PKC	protein kinase C
AT1	ANG°II type 1
cAMP	cyclic adenosine monophosphate
UTR	untranslated region
SFR	serum response factor
PDE	phosphodiesterases
CRE	cAMP-responsive elements
C/EBP	various CCAAT/enhancer binding protein
HADHB	hydroxyacyl-CoA dehydrogenase/3-ketoacyl-CoA thiolase/enoyl-CoA hydratase beta subunit
AMPK	AMP-activated protein kinase
