# Supplementary material for: Differential Regulation of Protective and Harmful Renin Transcripts by the cAMP/PKA/Ca2+-Pathway in Cardiac H9c2 Cells
Source: Cells. 2026 Jul 17;15(14):1281. doi: 10.3390/cells15141281 (PMC13406955; doi:10.3390/cells15141281)
Supplement: Supplementary file 1 [file cells-15-01281-s001.zip › cells-4388180-supplementary.pdf]

Differential Regulation of Protective and Harmful Renin Transcripts by the cAMP/PKA/Ca^{2+}-Pathway in Cardiac H9c2 Cells

Supplementary Materials:

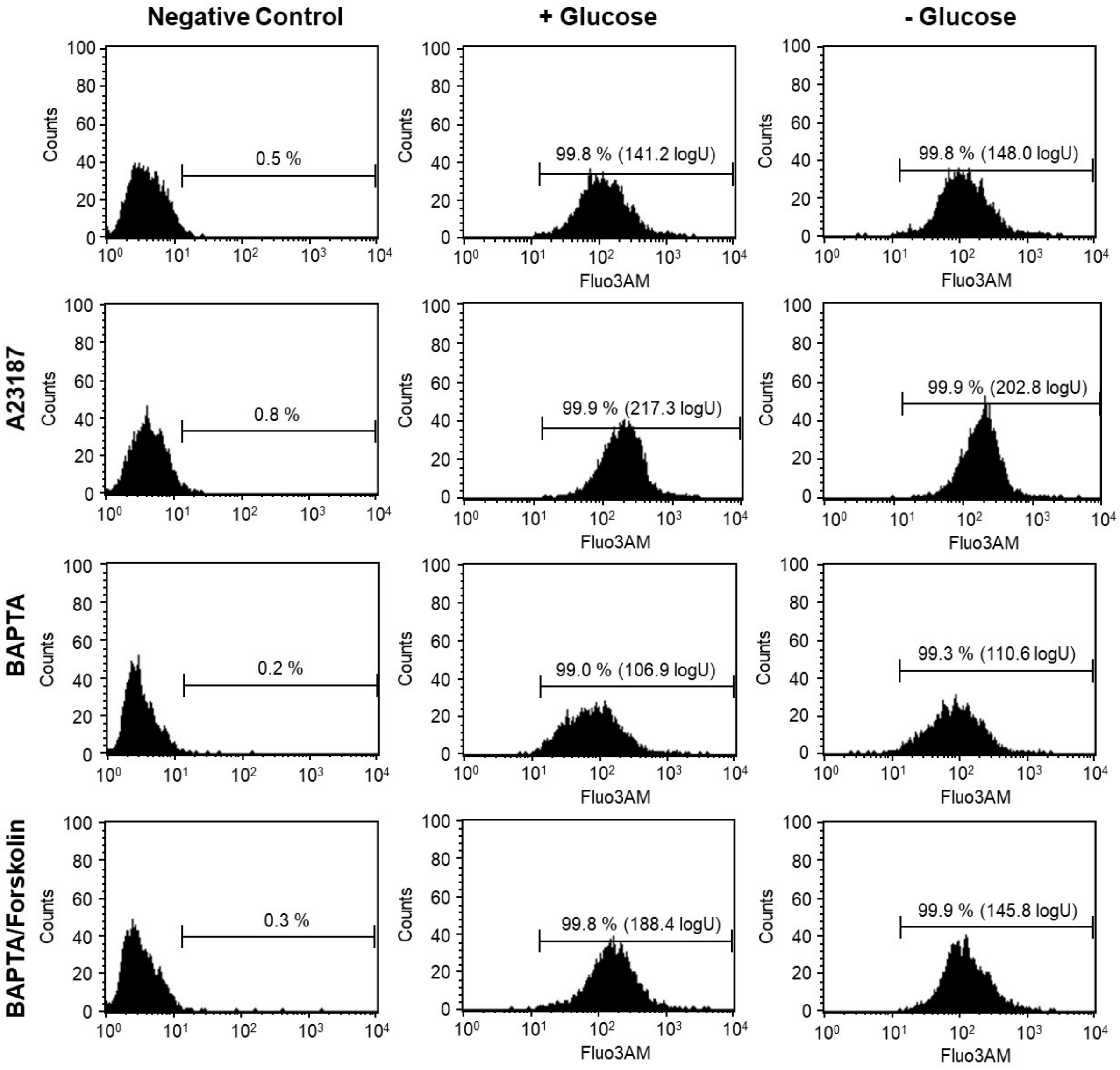

Figure S1. Effects of the calcium ionophore A23187 or the calcium chelator BAPTA on the free intracellular Ca^{2+} levels. For analyses, H9c2 cells were exposed to control conditions or glucose starvation alone or in combination with the calcium ionophore A23187 (1 µmol/L) or the calcium ions chelator BAPTA (50 µmol/L) without or with forskolin (10 µmol/L) for 24 h. The intracellular calcium content ([Ca2+]i) was monitored by flow cytometry using the Ca^{2+}-sensitive fluorophore Fluo3AM. Data show representative FACS images that include the

percentage of Fluo3AM-positive H9c2 cells and their mean fluorescence intensity (logU).

Table S1. Relative basal abundance of renin-a and renin-b transcripts in cardiac H9c2 cells. Transcript levels were determined by qRT-PCR and normalized to the reference gene YWHAZ (ΔCT). A lower ΔCT corresponds to higher transcript abundance. Values are given as mean ± standard deviation; n indicates the number of independent biological experiments.

Transcript	Condition	ΔCT (mean ± SD)	n
renin-a	+ glucose	15.41 ± 0.90	20
renin-a	– glucose	15.49 ± 0.96	20
renin-b	+ glucose	14.49 ± 0.82	14
renin-b	– glucose	12.97 ± 1.08	14

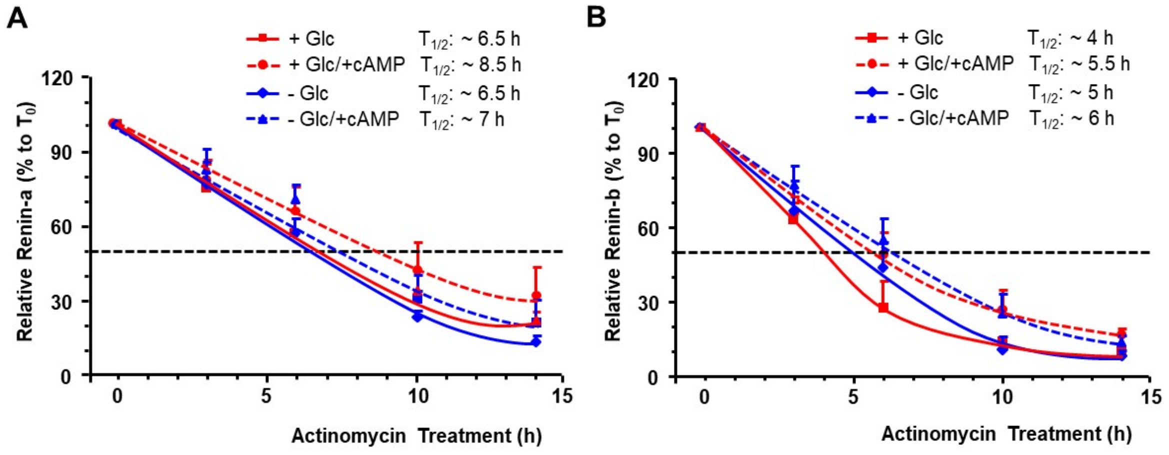

Figure S2. Half-lives of renin-a and renin-b mRNAs. Analyses were performed on H9c2 cells exposed to glucose starvation without or with cAMP supplementation, as indicated. (A) Half-life of renin-a and (B) half-life of renin-b transcripts after inhibition of RNA synthesis by actinomycin D at the indicated time points. Data represent mean ± SEM values from 3 independent experiments.
